# Extensive Arterial Dissection: A Rare Multivascular Emergency

**DOI:** 10.7759/cureus.76668

**Published:** 2024-12-31

**Authors:** Eduardo Macedo, Ana S Ramoa Oliveira, Martinha M Vale, Joana Lopes, Ana Rita Marques

**Affiliations:** 1 Internal Medicine, Hospital de Braga, Braga, PRT

**Keywords:** aorta surgery, arterial dissection, cardiothoracic and vascular surgery, cardiovascular disease, emergency medical service

## Abstract

Aortic dissection is a life-threatening vascular emergency associated with high morbidity and mortality. Clinical manifestations might include severe chest pain to neurological deficits, depending on the arterial segments involved. Extensive dissections involving multiple aortic segments and branch vessel occlusions, such as the carotid arteries, are rare and pose unique diagnostic and therapeutic challenges. We describe the case of a 64-year-old male, an active smoker with obesity, who presented to the emergency department with altered consciousness. He was found to have an extensive aortic dissection involving the ascending, arch, descending, and abdominal aorta, as well as occlusion of the right carotid artery. An urgent combined cardiothoracic and vascular surgical approach was undertaken. Despite suffering an ischemic stroke, the patient recovered successfully after a comprehensive rehabilitation program. This case underscores the importance of early recognition and a multidisciplinary strategy in managing extensive aortic dissection with neurological compromise. It highlights the potential of innovative surgical techniques and tailored rehabilitation to optimize outcomes in these complex cases.

## Introduction

Aortic dissection arises from a tear in the intima of the aortic wall, creating a false lumen that can propagate and compromise perfusion to vital organs, including the heart, brain, and kidneys. The condition is often classified by the Stanford system, with Stanford type A involving the ascending aorta, carrying particularly high mortality if left untreated [1]. Hypertension is a well-known risk factor, though other contributors such as smoking accelerate atherosclerosis and promote vascular inflammation, weakening the aortic wall [2]; obesity, which is associated with hemodynamic and inflammatory changes that may compromise aortic wall integrity [3]; connective tissue disorders and congenital valvular anomalies also play key roles [4,5].

The clinical presentation can be atypical, particularly when the dissection extends beyond the arch and involves branches such as the carotid arteries. Neurological deficits may be the initial manifestation, delaying recognition and increasing risk [6]. Early diagnosis is crucial, as emergency surgical or endovascular intervention can be lifesaving. We present a case of an extensive aortic dissection involving multiple aortic segments and the right carotid artery, resulting in neurological compromise, and discuss the surgical strategy, rehabilitation, and key lessons learned.

## Case presentation

A 64-year-old man, an active smoker (20 pack-year) and obese (BMI of 31 kg/m2), was admitted to the emergency department in an acute confusional state. The initial evaluation revealed hemodynamic instability, characterized by hypotension with a systolic pressure of 87 mmHg and a diastolic pressure of 55 mmHg and with tachycardia at 130 bpm, prompting further diagnostic investigation. The initial workup ruled out acute myocardial infarction (with consistently negative myocardial necrosis markers), infectious etiology (as C-reactive protein levels were negative and no leukocytosis was observed), and acute alcohol intoxication.

**Figure 1:**
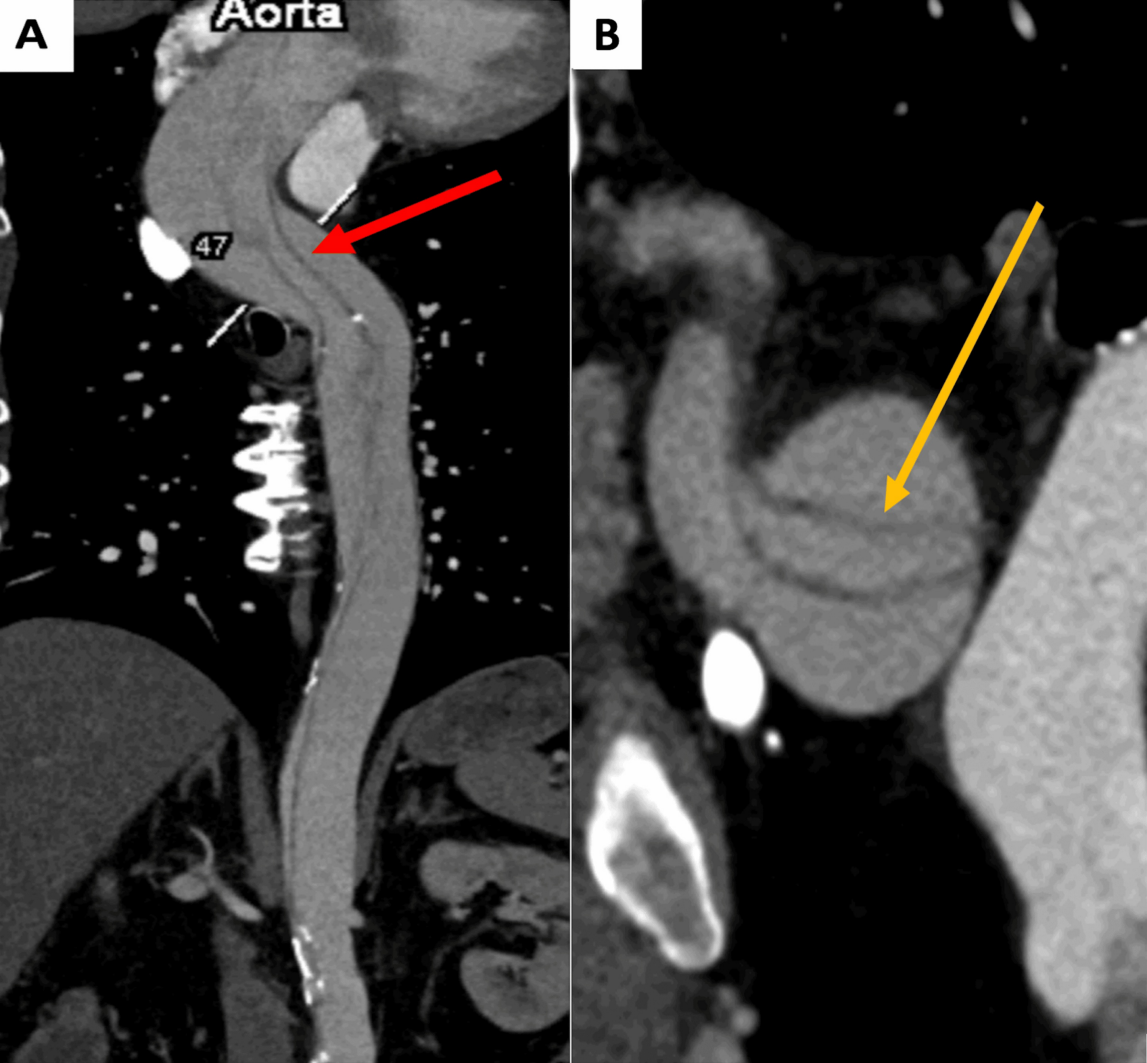
A-B: CT angiography reconstructions revealing an extensive aortic dissection involving the ascending, arch, descending thoracic, and abdominal aorta (A) Longitudinal extent of the dissection (marked with the red arrow). (B) Cross-sectional view demonstrating the separation between the true and false lumens (marked with the yellow arrow). CT: computed tomography

**Figure 2:**
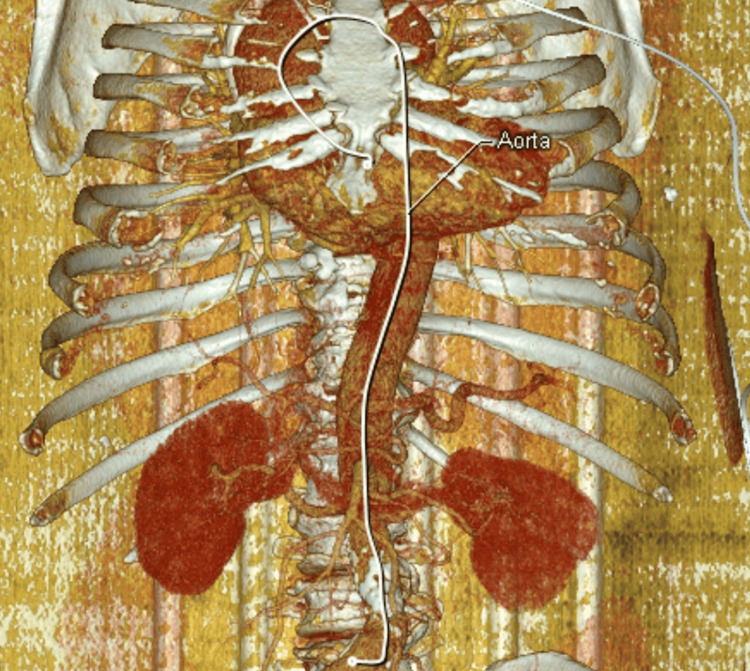
3D CT angiography reconstruction showing an extensive aortic dissection involving the ascending, arch, descending, and abdominal aorta The extent of the dissection is marked with the white line identifying the aorta. 3D: three dimensional, CT: computed tomography

**Figure 3:**
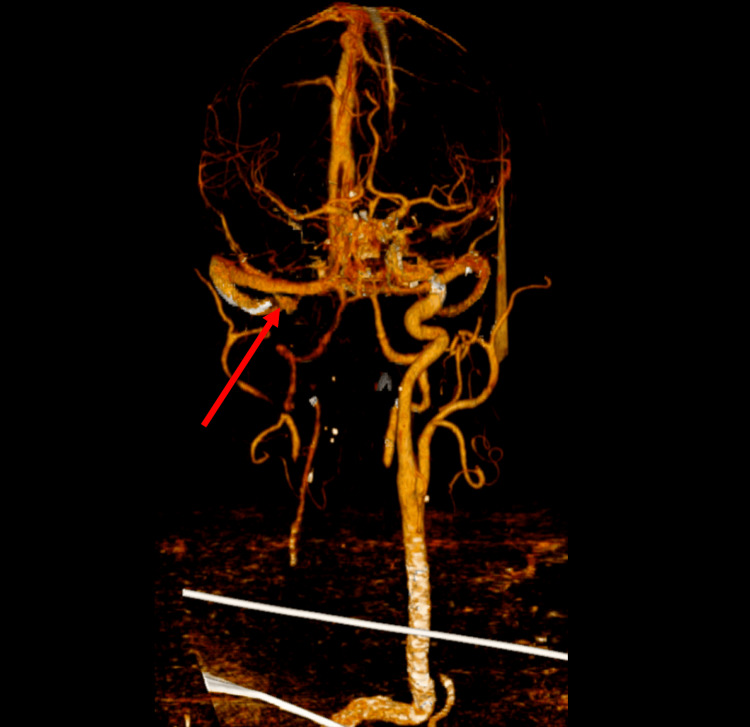
3D CT angiography reconstruction of the head and neck vessels demonstrating a right carotid artery occlusion alongside the arterial branches supplying the brain The occlusion is marked by the red arrow. 3D: three dimensional, CT: computed tomography

An urgent contrast-enhanced CT (angiography) was performed, revealing an extensive arterial dissection that extended through the ascending aorta, arch, descending aorta, and abdominal aorta (Figure 1 and Figure 2), as well as occlusion of the right carotid artery (Figure 3).

A Stanford type A aortic dissection extending beyond the arch was confirmed, and the patient was submitted to surgery with cardiothoracic and vascular surgeons. The surgical team replaced the ascending aorta and part of the arch with a synthetic graft, while an endovascular repair was employed for the descending and abdominal aortic segments. Postoperatively, the patient experienced an ischemic stroke due to right carotid occlusion that was not previously completely treated due to the extent of the thrombus and the potentially fatal damage. However, the aortic repair was successful, and following an intensive physiotherapy program, he demonstrated progressive recovery of neurological function. The patient remained hospitalized for two weeks in the intensive care unit and a total of one and a half months in the hospital. At the six-month follow-up, the patient remained stable, with no further hemodynamic compromise and improved motor abilities.

## Discussion

Extensive aortic dissections involving the ascending, arch, descending, and abdominal segments, as well as supra-aortic branches such as the carotid arteries, are rare and associated with a high risk of mortality and morbidity [1]. The most common risk factor for aortic dissection is hypertension, which increases wall stress and leads to intimal tears [2]. Smoking is associated with endothelial dysfunction, chronic inflammation, and accelerated atherosclerosis, all of which predispose to arterial damage [5]. Obesity is also known to increase inflammatory mediators and oxidative stress, further contributing to cardiovascular disease [3,7]. In this patient, chronic smoking and obesity were probably the major factors. Although hypertension was not explicitly reported, it remains a common comorbidity in aortic dissection, especially in the early stages of the disease.

Aortic dissection presents with abrupt, severe chest pain described as tearing. However, neurological symptoms or syncope might be the only presenting symptoms in some patients, potentially delaying the diagnosis [6]. In the case described, altered consciousness and hemodynamic instability raised suspicion of a catastrophic cardiovascular or cerebrovascular event, prompting the diagnostic CT angiography that ultimately confirmed the dissection.

For Stanford type A dissections, urgent surgical repair is necessary to prevent cardiac tamponade, rupture, and death. Hybrid procedures, combining open surgery for the ascending aorta and arch with endovascular techniques for the descending segments, have gained traction for managing complex anatomical involvement [8].

Despite developing an ischemic stroke, the patient in this case achieved a positive surgical and neurological outcome with comprehensive postoperative care, including targeted physiotherapy [9]. Close neurological assessment and integrated care by cardiothoracic, vascular, neurology, and rehabilitation teams are crucial to optimizing patient prognosis [10].

## Conclusions

This case illustrates the severity and complexity of extensive aortic dissection involving multiple segments of the aorta and the right carotid artery. This case highlights the importance of early diagnosis based on high clinical suspicion and prompt imaging studies and the role of a multidisciplinary surgical team in managing this condition. Recognizing and addressing modifiable risk factors, such as smoking and obesity, remain essential strategies for preventing cardiovascular events. Long-term follow-up is recommended to monitor the integrity of the surgical repair and any other complications that may occur, such as hypotension and shock, recurrent pain, pericardial effusion and tamponade, periaortic hematoma, brain injury, or ischemia.
